# Addressing Tobacco-Related Disparities Among Youth Experiencing Homelessness by Engaging Youth Collaborators in Intervention Research: Protocol for a Multimethod, Community-Based Participatory Research Study

**DOI:** 10.2196/69441

**Published:** 2025-08-19

**Authors:** Alana R Lopez, Andrew C Lim, Renatta Escobedo, Nicole Chung, Lorilee Chien, Jerel P Calzo, Lianne Urada, Benjamín Aceves, Steven Jellá, Sabrina L Smiley, Jennifer K Felner

**Affiliations:** 1 Institute for Behavioral and Community Health San Diego State University Research Foundation San Diego, CA United States; 2 Joint Doctoral Program in Public Health San Diego State University and University of California, San Diego San Diego, CA United States; 3 San Diego Youth Services San Diego, CA United States; 4 School of Public Health San Diego State University San Diego, CA United States; 5 School of Social Work San Diego State University San Diego, CA United States; 6 Department of Medicine University of California, San Diego San Diego, CA United States

**Keywords:** youth engagement, intervention research, tobacco disparities, homelessness, runaway and homeless youth, youth advisory board, youth action board

## Abstract

**Background:**

Youth experiencing homelessness and housing instability exhibit disparities in tobacco use and co-use of tobacco and cannabis products compared to their stably housed peers. Interventions to address these disparities have had limited success to date. This may be related to a lack of fit between intervention approaches and the unique needs and preferences of this marginalized population. Leveraging strategies to facilitate authentic engagement of youth in intervention research, including collaborating with youth in the cocreation of intervention goals and strategies, presents an opportunity to overcome challenges of previous research to mitigate tobacco-related disparities among youth experiencing homelessness and housing instability.

**Objective:**

This protocol aims to engage youth collaborators in intervention research to address tobacco-related disparities among youth experiencing homelessness and housing instability. It outlines strategies to recruit and partner with youth to cocreate a strengths-based, expressive arts intervention to reduce harms associated with tobacco use and tobacco/cannabis co-use and evaluate our youth engagement strategies.

**Methods:**

The community and academic partners leading this intervention study developed a structure that allows for two levels of youth collaboration. This structure, developed through consultation with a youth action board, facilitates youth (aged between 14 and 26 years) involvement in intervention design, implementation, and pilot testing. At the higher collaboration level, youth will join the intervention research team as “co-researchers,” engaging weekly with study-related tasks and contributing equitably to the research processes (eg, selecting intervention strategies and supporting data analysis) in alignment with their personal and professional goals. At the lower collaboration level, youth will engage with the research team as “advisors,” providing targeted feedback at key points during intervention development. Brief surveys, field notes, ethnographic memos, focus group discussions, and semistructured interviews will be used to evaluate our process and impact among the engaged youth.

**Results:**

To date, we have engaged 7 co-researchers who have participated in several capacity-building and professional development activities, including research ethics training and tobacco prevention advocacy training. Co-researchers have contributed to intervention development brainstorming sessions, evaluated available interventions to address youth tobacco use, and developed materials to expand the group of co-researchers and advisors. Preliminary data suggest sufficient youth engagement (study goal of engaging 10 co-researchers in a 2-year study period) and that engagement processes are highly acceptable to youth collaborators. Lessons learned to date have helped us to improve and refine our engagement processes (eg, technological barriers).

**Conclusions:**

This protocol describes a holistic approach to equitably and authentically engaging youth as collaborators in intervention research to address tobacco-related disparities among youth experiencing homelessness and housing instability. The approach may serve as a useful model that can be adapted for other youth-focused intervention research.

**International Registered Report Identifier (IRRID):**

DERR1-10.2196/69441

## Introduction

### Background

Cigarette smoking and secondhand smoke remain the leading causes of preventable death and illness worldwide and in the United States [1-4], where nearly 1 in 5 deaths is caused by smoking or exposure to secondhand smoke [4]. Despite significant decreases in smoking over time, particularly in the last 2 decades [5], more than one-fifth of adults and one-tenth of adolescents in the United States report past-month tobacco use in various forms (including cigarettes, e-cigarettes, and other combustible and smokeless tobacco products) [6,7].

### Tobacco and Cannabis Prevalence and Health Impacts Among Youth

Smoking and other tobacco use have negative health implications for people of all ages; however, tobacco use in adolescence and young adulthood can negatively impact health across the life course. For example, most adults who smoke tried their first cigarette by the age of 18 years, with a mean age of smoking initiation of approximately 15 years for those who smoke daily [3]. Early initiation of tobacco use is associated with the development of substance use disorders and respiratory, cardiovascular, and other physical health problems in both youth and adults [8]. Tobacco use in adolescence and young adulthood can also have harmful effects on brain development related to learning, mood, impulse control, and academic achievement [8-10]. While cigarette smoking has decreased dramatically for youth over time, rates of e-cigarette use (ie, “vaping”) have largely increased in recent years [11]. Though there is some indication that vaping among adolescents is beginning to decline for the first time in a decade [6], vaping among young adults has continued to increase [12]. As vaping increases, so does vaping dependence. For example, research has found that current levels of vaping dependence among individuals aged between 16 and 19 years in the United States, Canada, and England are comparable to or higher than rates of cigarette dependence, with between 45% and 50% of youth who vape reporting experiences and behaviors consistent with vaping dependence [13].

Similar to tobacco use, cannabis use is associated with negative health impacts among youth, including increased risk for other substance use and disorders [14,15]; impairments to cognitive functioning [16,17]; and mental health problems, such as depression, anxiety, and other psychiatric disorders [15,18]. Among adolescents, cannabis use has remained relatively stable or even decreased over time, with approximately 17% of the adolescents reporting past 30-day use in 2023 compared to 23% in 2013 [19], with some of the largest 1-year decreases ever recorded occurring from 2020 to 2021 (during the COVID-19 pandemic) [20]. These trends are reversed among young adults, however, with significant increases over the last several decades in the number of young adults who report regular cannabis use [21]. For example, among young adults aged 19 to 30 years, participating in the 2023 Monitoring the Future panel study, 29% reported past-month cannabis use, with the highest prevalence of 32% among young adults aged between 23 and 24 years [21]. These rates are among the highest levels of cannabis use ever recorded, which is a 10% increase since 2013.

Tobacco/cannabis co-use (ie, simultaneous use or use of both products within the same time period) is increasingly common among adolescents and young adults, and in some studies, has been found to be more common than using 1 substance only [22,23]. Co-use has also been found to be associated with more frequent and higher quantities of use of both products [24] and increased risk for mental health problems, respiratory symptoms, and lower academic achievement [22,25-27]. Tobacco and cannabis use among young adults share many common determinants, including the use of substances to cope with emotional or psychological distress, peer influence, and the availability of substances [28,29]. However, some distinct drivers of cannabis use include beliefs that cannabis is less harmful to health than tobacco and that it may help to tune out of or escape from one’s environment [29,30]. Importantly, emerging evidence points to frequent cannabis use as a barrier to tobacco cessation [31]. This highlights the need to account for dual use of tobacco and cannabis in efforts to reduce use of either substance, as a failure to do so may limit the effectiveness of these efforts [28,30,32].

### Tobacco-Related Health Disparities: A Focus on Youth Experiencing Homelessness and Housing Instability

While smoking and other tobacco use has decreased for some groups over time, the US surgeon general’s recent report on tobacco-related morbidity and mortality highlights persistent tobacco-related disparities along axes of identity and social position, including race or ethnicity, income, education, sexual orientation, gender identity, occupation, geography, and behavioral health status [4]. The report refers to these disparities as a “social injustice, in addition to an economic and health burden” [4]. Among the populations identified by the report as evidencing persistent tobacco-related disparities are people experiencing homelessness who have been specifically targeted by the tobacco industry through marketing and efforts to thwart laws that would promote smoke-free policies in housing programs.

A group of particular concern with respect to addressing tobacco-related health disparities across the life course is youth experiencing homelessness and housing instability. Indeed, youth experiencing homelessness and housing instability exhibit rates of tobacco use that are markedly higher than their stably housed peers. Previous research has found that as many as 70% of the youth experiencing homelessness and housing instability smoke and use other tobacco products (eg, e-cigarettes), which is significantly higher than the general population of adolescents and young adults nationally [33-36]. Rates of cannabis use among youth experiencing homelessness and housing instability are similarly high, with estimates that nearly 60% use cannabis [37], which is nearly 2 to 3 times the rate of their stably housed peers [38,39]. Youth experiencing homelessness and housing instability who smoke cigarettes also tend to engage in harmful smoking-related behaviors, such as smoking discarded cigarettes, blocking filter vents, and rolling cigarettes with tobacco from discarded cigarette butts [40,41]. Emerging evidence has begun to find high rates of coconsumption of tobacco and cannabis among youth experiencing homelessness and housing instability as well [42,43]. Importantly, coconsumption may result in more negative impacts on youth development and opportunities for financial stability and social mobility than tobacco use alone. These tobacco- and cannabis-related disparities are a significant health concern for a group particularly susceptible to concomitant negative health and social outcomes [33,44,45].

### The Need for Youth-Engaged Intervention Research

Despite documented tobacco-related disparities among youth experiencing homelessness and housing instability, there is evidence that many are interested in quitting or reducing their tobacco use [46-48], and service providers working with this population are committed to supporting their desired behavior changes and goals [49,50]. There have been relatively few intervention studies that aim to address tobacco-related disparities among youth; however, there have been several studies focused on adults experiencing homelessness. To date, these interventions, for both youth and adults, have demonstrated limited to moderate success. For example, a 2020 systematic review of 10 randomized controlled trials of tobacco cessation interventions for adults experiencing homelessness (aged >18 y) found mostly inconclusive evidence to assess intervention effects [51]. A tobacco cessation intervention for youth experiencing homelessness and housing instability using mobile health approaches (text messages) found small to moderate effect sizes [32,52]. A 2013 systematic review of 8 peer-support programs for smoking cessation in “disadvantaged groups,” including people experiencing homelessness, found limited evidence of efficacy [53]. Several potential mechanisms of change [54] have been identified and examined across these intervention studies, including increasing access to peer support [32,52,53], enhancing motivation to reduce tobacco use [55,56], and reducing emotional distress [57,58]. Among these, peer support has emerged as a particularly promising approach [53], although overall intervention effects have been variable. This points to a need for a deeper examination of the barriers limiting intervention efficacy in this population.

Several factors contributing to the limited efficacy of previously tested interventions have been identified, including limited statistical power, limited intervention follow-up, and a lack of fit between available interventions and the needs of people experiencing homelessness. Another potential factor includes a lack of intervention fit with the community-based contexts where people experiencing homelessness access resources and build community [51,53]. This lack of fit may be related to a lack of meaningful engagement of people with lived experience of homelessness (as well as the individuals working within service provision contexts) in the intervention development process. Although several published intervention studies indicate gathering insights from relevant stakeholders to inform interventions (eg, 1-time interviews or focus groups), few, if any, explicitly note the inclusion of people with lived experience of homelessness as research collaborators throughout the development and implementation of interventions.

For intervention research focused on addressing tobacco-related health disparities among youth, leveraging strategies to facilitate authentic engagement of youth in the research process, including in the cocreation of intervention goals and strategies, presents an opportunity to overcome challenges of previous research in this area. Indeed, a recent review of behavioral health interventions for youth experiencing homelessness and housing instability found that of 33 unique interventions, fewer than half involved youth or community stakeholders in the design of the intervention, and among those, even fewer involved youth as sustained collaborators in the intervention research process [59].

### Objectives

The objective of this protocol is to provide an overview of our youth engagement process and outline strategies to promote equitable collaboration with youth in the context of an intervention pilot study to address tobacco-related disparities among youth experiencing homelessness and housing instability. In addition, we aim to delineate our strategy to evaluate our approach to youth engagement in terms of process and outcomes among youth collaborators.

## Methods

### Overview

Tobacco and Cannabis Harm Reduction through Expressive Arts (TobExA) is a community-based participatory research pilot study involving the development, implementation, and pilot testing of an intervention to address tobacco-related disparities among youth experiencing homelessness and housing instability. The study is guided by harm-reduction and strengths-based frameworks.

Aligned with the principles of community-based participatory research, TobExA is equitably coled by investigators at a community-based organization serving youth experiencing homelessness and housing instability (“community partner”) in San Diego County, California, and a local public state university (“academic partner”). Previous research has documented the utility of community-academic collaborations in the creation of interventions to mitigate health disparities [60,61], with greater and more sustained impacts than researcher-driven interventions that are not cocreated with community partners [62,63].

The initial idea for TobExA was based on the community partner organization’s priority of addressing health disparities and mitigating health risk behaviors among their youth clients accessing emergency shelter and drop-in spaces. Particularly, administrators and staff indicated an interest in understanding the influence of substance use, including tobacco and cannabis product use, on the health and stability of the youth they serve, and addressing tobacco-related disparities through harm reduction and strengths-based approaches. Harm reduction and strengths-based approaches are aligned with the community partner organization’s mission and priorities and have been identified as evidence-based strategies for addressing substance use and other health disparities affecting people experiencing homelessness [44]. In addition, several members of the community partner organization’s staff have expertise in expressive arts therapy and regularly use expressive arts techniques to engage their youth clients.

The TobExA intervention development process is guided by intervention mapping (IM), a standardized, yet flexible 6-step framework for building theory- and evidence-based health promotion interventions [64]. IM is inherently community engaged, emphasizing the need for feedback from diverse partners at each point in the intervention research process. TobExA partners include the community and academic partner organizations coleading the study, service providers and administrators serving youth experiencing homelessness and housing instability across the San Diego region, and youth with lived experience of homelessness or housing instability.

We assert that authentic youth engagement is central to effectively developing an intervention that will center the needs, experiences, and preferences of youth. This paper outlines our protocol for the engagement of youth collaborators in the TobExA study, including in the development of the intervention. Of note, a protocol detailing the TobExA intervention will be forthcoming (intervention development is in progress). Briefly, the TobExA intervention will be a multilevel group-based intervention grounded in harm reduction and strengths-based frameworks, with expressive arts as a key modality of participant engagement and reflection. The intervention will prioritize goal setting and social support as theory-based strategies for promoting reductions in tobacco and cannabis use among youth experiencing homelessness. The intervention will be co-designed with youth collaborators (as discussed herein).

### Guiding Theoretical Framework: Youth Participatory Action Research

The TobExA youth engagement procedures outlined in this protocol are guided by a youth participatory action research framework. Informed by emancipatory education and research [65], youth participatory action research situates young people as “legitimate and essential collaborators” in research and action on issues affecting their lives [66]. Youth participatory action research projects are typically situated within educational or community-based program settings and focus on the needs of marginalized or minoritized communities of youth [67]. Because youth understand their lives in ways that adults cannot, engaging youth as equitable collaborators in the intervention research process will support the development and implementation of an intervention with improved fit and impact than an intervention developed by adult researchers alone [68]. Indeed, youth involved in the cocreation of interventions experience benefits from their participation (eg, personal and professional skills), and youth who participate in interventions that are cocreated by youth experience positive intervention impacts. Thus, there is an individual and community benefit to youth engagement and collaboration in intervention research [69,70].

Youth engagement as a construct captures a broad range of youth participation in the research process. For example, “engagement” in some intervention research may be characterized by relatively low levels of sustained youth involvement or leadership, such as youth providing one-time feedback to inform an intervention during development. In other research, “engagement” may involve youth as collaborators and decision makers throughout the research process, with youth serving as members of the research team whose perspectives are given equal weight to other collaborators, such as investigators, consultants, etc [71]. Although youth engagement has been identified as an “ethical imperative” [72] and “principle in care” [73] in the development of interventions to address youth substance use, there are many challenges to authentic and ethical youth engagement in research. These challenges often relate to power differentials between youth collaborators and adult researchers and limited opportunities for youth to be involved in shared and equitable decision-making [72,74,75].

### Youth Engagement Overview

In the spirit of youth participatory action research and to overcome the challenges of previous youth-engaged research, our youth engagement approach and specific processes have been developed through multiple consultations with youth with lived experience of homelessness or housing instability as well as staff serving youth experiencing homelessness and housing instability. Specifically, members of the TobExA research team engaged members of the community partner’s youth action board (YAB) at the beginning of the study to gather feedback on in-development plans for youth engagement (eg, compensation, expectations, style of engagement, and recruitment strategies). Although we discussed collaborating with the existing YAB as the primary youth engagement strategy for the TobExA study rather than initiating a study-specific youth engagement protocol, YAB members indicated that study-specific engagement was necessary so as not to compete with the YAB’s current priorities.

TobExA’s youth engagement processes will involve members of the research team, including investigators, research assistants, and other support staff, collaborating with youth as “co-researchers” or “advisors” throughout the intervention research process. They will be invited to commit 5 to 10 hours to collaboration activities (ie, research tasks) per week for a minimum of 3 months (with the opportunity to extend collaboration for the duration of the study). Co-researchers will be recruited on a rolling basis to ensure approximately 2 to 5 active co-researchers throughout the project timeline. Co-researchers will participate in 1 or more stages of intervention design, pilot testing, and dissemination of findings, depending on project timing at recruitment and length of co-researcher engagement. Advisors will provide targeted feedback during the intervention development process via synchronous and asynchronous modalities on 1 or more occasions.

### Eligibility and Recruitment

We will recruit approximately 10 co-researchers and 10 advisors across the 2-year study period (August 2023 to July 2025). To be eligible, prospective co-researchers and advisors must be aged between 14 and 26 years, have lived experience with homelessness or housing instability, and be fluent in English or Spanish. They must also be willing to commit to abstaining from using substances (including tobacco, cannabis, alcohol, and other drugs) during their *active* hours contributing to the TobExA study; there are no requirements or expectations regarding abstaining from substance use outside of these active contribution periods. The decision to require abstinence during active hours is aligned with the harm reduction focus of TobExA (as opposed to abstinence or complete cessation of substance use) and our goal of developing the professional capacity of youth to support their success in other professional settings.

Co-researchers and advisors will be recruited via direct referrals, word of mouth, and in-person presentations. Recruitment efforts will focus on the following groups of youth: (1) members of the community partner’s YAB, (2) youth receiving services from the community partner organization who are not members of the YAB, and (3) youth receiving services at other local organizations serving youth experiencing homelessness and housing instability. Interested youth will communicate their desire to join the study as collaborators via a study-specific GroupMe channel (Microsoft Corporation), Google Form, email, or a community partner organization staff member’s direct referral. Recruitment will be ongoing throughout the study period, as needed. We anticipate focused recruitment “pushes” will occur at key time points in the IM process to support phase-specific youth engagement (eg, during program development and implementation planning).

### Youth Engagement Procedures

#### Overview

TobExA’s youth engagement processes will be led by “youth engagement liaisons” (including the lead coauthors of this protocol, who are current doctoral students [ARL and ACL]) with supervision from the academic co–principal investigator (JKF) and a community partner coinvestigator (RE).

#### Co-Researchers

After expressing their interest in collaborating with the TobExA team, prospective co-researchers will participate in a preliminary onboarding interview (approximately 1 hour) to assess their personal and professional development goals, research interests, current skills and strengths, and availability. Those who are able to commit approximately 5 to 10 hours per week for 3 months (with flexibility as needed) and whose interests are aligned with the study or research generally will be invited to join the study as co-researchers. Before being assigned research tasks, co-researchers will participate in a group training on human research protections and receive an in-depth introduction to the TobExA study. Co-researchers will then be assigned weekly tasks based on study needs and their individual interests and goals. They may also begin to attend TobExA study team meetings at this time.

Co-researchers will participate in 30-minute biweekly individual check-in meetings with the youth engagement liaisons to discuss tasks and troubleshoot any issues or concerns. Every 2 weeks, all co-researchers will participate in a 1.5-hour group meeting led by the youth engagement liaisons where co-researchers will share updates on their tasks; build community with fellow co-researchers and youth engagement liaisons; and collaborate on research tasks, such as analyzing interview data to inform the TobExA intervention (with guidance from the youth engagement liaisons). Co-researchers may complete most of their assigned tasks remotely (with many tasks designed to be completed on a smartphone if a computer is not easily accessible), with group meetings predominantly held in person. They will have access to workspaces with computers and internet at the community and academic partners’ offices.

We acknowledge that substantial attrition among co-researchers may occur during the expected 3-month engagement period, partly due to their developmental and life stage (characterized by educational, familial, and personal transitions and changes) and the many stressors and competing priorities for youth with current or previous experiences of homelessness or housing instability [76-78]. To support the retention of co-researchers, we will monitor attendance at weekly group and individual meetings. If waning engagement is noted, youth engagement liaisons will work collaboratively with youth and community partners to assess the individual needs of co-researchers and determine the most appropriate support strategies. These may include additional development opportunities or, if necessary, a compassionate offboarding process. Exit interviews and periodic focus group discussions (detailed subsequently) will provide additional insights into reasons for disengagement and co-researcher retention strategies and will be used to drive changes to strategies as needed. This structured process, along with the co-researcher role’s co-designed nature between youth, academic, and community partners, is anticipated to promote engagement in the long term. Several of the authors have experience collaborating with youth in other youth participatory action research studies in which study goals were achieved with as few as 2 to 4 highly involved youth collaborators [79,80]. Thus, even if we experience high rates of attrition among co-researchers, we expect to engage a sufficient number of youth collaborators throughout the study period to meet our goals.

#### Advisors

As noted earlier, advisors will participate in specific opportunities to provide feedback on the TobExA intervention. These opportunities will be synchronous, such as group or individual meetings with study staff (in person or over Zoom), or asynchronous, such as providing feedback via email or a secure messaging app. Synchronous opportunities (eg, group meetings) will be coled by co-researchers who will develop the agenda, cofacilitate discussions, and take the meeting minutes. Advisors may participate in 1 or more feedback opportunities (we expect to hold a minimum of 2 feedback opportunities during the study period) and will receive US $19 per hour for their participation, with additional stipends to cover transportation and childcare as needed. Advisors will be given (in person) or mailed a check after participating in a feedback opportunity.

#### Youth Engagement by Research Stage

Multimedia Appendix 1 provides a visual representation of the TobExA intervention research process (including the 6 steps of IM [64]), crosswalked with youth collaborator participation time points and activities. The first phase of IM (step 1) is the *needs and assets assessment*, which involves developing a *logic model of the problem*. During this phase, we will conduct formative interviews with stakeholders and youth to assess reasons for tobacco and cannabis use among youth experiencing homelessness as well as needs to prevent and reduce use in this population (ie, needs-assessment phase). We will also focus on assessing the context in which the intervention will be implemented to identify strategies to support intervention fit and sustainability. Although co-researchers will not be involved in data collection for this initial phase of the research process, they will provide feedback on and contribute to the development of the *logic model of the problem* during group meetings led by the youth engagement liaisons. These early contributions will be essential to ground the IM process in the local context as informed by the lived experiences and community knowledge of co-researchers and other stakeholders who are familiar with the local context (through lived experience, work roles, etc).

Co-researcher and advisor engagement will be especially critical during the subsequent stages of the research process, including development of the intervention (IM steps 2-4), planning for implementation and evaluation (IM steps 5-6), and piloting the intervention. Throughout these stages, co-researchers and advisors will provide synchronous and asynchronous input that will be directly incorporated into the intervention research process. Specifically, co-researchers will participate in several structured activities to ensure their equitable contributions to the intervention development process. This includes participating in analysis of qualitative data gathered during IM step 1, reviewing and providing feedback on materials to consider for inclusion in the intervention (eg, existing tobacco intervention materials and resources), and attending intervention development meetings with the full TobExA team. Co-researchers will also be invited to lead presentations for the TobExA team to share their ideas and recommendations for the intervention. Co-researchers will be involved in piloting the intervention (eg, supporting recruitment, data collection, and cofacilitation) and supporting dissemination of findings (eg, via scientific conferences, community-based presentations and events, and fact sheets).

Co-researchers will lead discussion sessions or asynchronous communication with advisors to gather their feedback during 2 key phases of the IM process (steps 3-4 and 5-6). Advisors will be asked to respond to specific open-ended prompts or discussion questions that will shape decisions regarding the intervention content and implementation strategies. For example, we may gather feedback from advisors to identify the best approach to hosting intervention group sessions based on age, with questions such as, “The intervention will include group sessions. If you were attending a group about reducing tobacco and/or cannabis use, what age range would you feel most comfortable with? Why?” Advisors will also be invited to provide feedback on recruitment strategies and other related considerations for piloting the intervention, such as appropriate incentives for participation, strategies for data collection, and strategies for gathering detailed participant feedback. Co-researchers will summarize and share feedback from the advisors with the full TobExA team to ensure advisors’ contributions are meaningfully integrated into the intervention research process (at TobExA team meetings or via asynchronous communication). Although we do not currently have plans to engage advisors in findings dissemination, we may schedule feedback opportunities as part of findings dissemination to “member check” our interpretations and gather recommendations for next steps in the TobExA intervention research process (eg, how to improve the intervention for a future efficacy trial).

### Measures and Tracking

#### Overview

Multiple strategies will be implemented to evaluate and document youth engagement strategies and processes throughout the study. To document the process of engagement and identify opportunities for quality improvement, youth engagement liaisons and other TobExA team members working directly with youth collaborators will write field notes and ethnographic memos based on their experiences and learnings (yielding qualitative data).

We will collect data from youth collaborators to document sociodemographic characteristics of youth engaged (co-researchers and advisors—age, education, race, ethnicity, sex assigned at birth, gender identity, transgender status, sexual orientation, and recent housing experiences), evaluate the impact of collaboration on the personal and professional development of youth (co-researchers only), and identify opportunities for quality improvement in our engagement processes (co-researcher and advisors).

**Figure 1:**
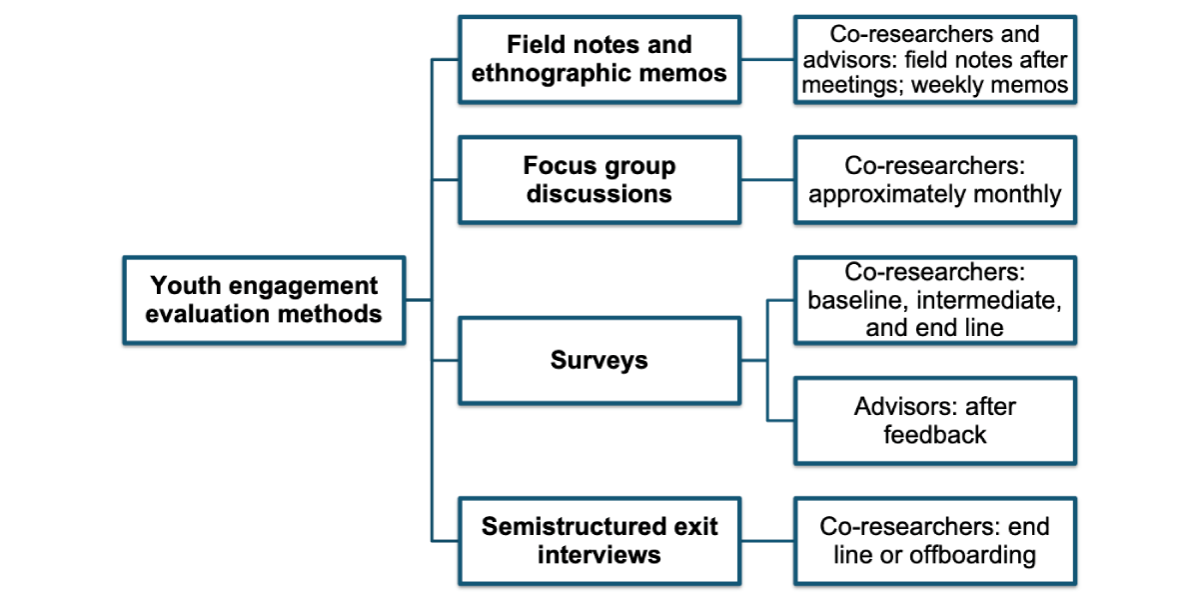
Youth engagement evaluation methods.

Co-researchers will complete surveys at multiple time points, participate in periodic focus group discussions, and complete a semistructured exit interview (approximately 45 min long) when they officially end their time as a co-researcher. Survey measures were pilot-tested with a subsample of co-researchers via “think-aloud” cognitive interviews (mean 105, SD 15 min), which allow youth to verbally discuss the survey questions and explain the factors contributing to their answers [81]. Advisors will complete short surveys after participating in a feedback opportunity. Surveys were piloted with members of the community partner YAB. Figure 1 presents a visual overview of evaluation methods. We further detail these evaluation strategies in the subsequent sections.

#### Field Notes and Ethnographic Memos

Youth engagement liaisons and other TobExA team members working directly with youth collaborators will write semistructured field notes after each biweekly meeting with the co-researchers and advisors and weekly unstructured ethnographic memos on the overall youth engagement and collaboration process [82]. Field notes will include prompts to narratively describe the following: (1) barriers to and facilitators of equitable and sustainable youth engagement and collaboration, (2) the application of key youth participatory action research and community-based participatory research principles to our youth engagement processes, and (3) how youth engagement and collaboration activities are connected to and facilitating the completion of TobExA study aims. Field notes will also capture noteworthy milestones and activities in co-researcher development, such as participation in major training and professional development opportunities.

Ethnographic memos will be in the form of unstructured narrative reflections regarding co-researcher–related activities and progress. Memos will document key challenges or needs in the youth engagement process and identify opportunities to refine youth engagement and collaboration strategies. Memos will be regularly reviewed by the academic co–principal investigator and other supporting coinvestigators, allowing for real-time or near real-time communication regarding emergent challenges to be addressed (ethical, logistical, and process related).

#### Youth Co-Researcher Surveys

We will administer surveys to co-researchers at multiple time points during their involvement in the study (baseline, intermediate, and end line). Baseline surveys will be administered upon the onboarding of youth into the project. Intermediate surveys will be completed iteratively between the defined steps of IM. End line surveys will be administered when youth begin offboarding from their co-researcher roles. The surveys will include items from validated scales and specific measures from the following surveys, scales, and subscales:

- Adult support, empowered skill-building, and positive values subscales from the Youth Program Quality Survey (α=.71, .77, and .79, respectively) [83]
- Supportive adult relationships subscale from the Youth-Adult Partnership Scale [84]
- Participatory behavior subscale from the Research and Action Self-Efficacy Scale (α=.83) [85]
- Self-efficacy or belief-in-self subscale from the Social Emotional Health Survey (α=.76) [86]
- Youth empowerment and leadership skill subscale from the Sociopolitical Control Scale for Youth [87]
- Youth voice in decision-making subscale from the Youth-Adult Partnership Scale [84]
- Perceived Control Scale—organizational and community levels (α=.61 and .63) [88]
- Flourishing individuals subscale from the Community Well-Being Assessment [89]

As noted earlier, we pilot-tested an initial version of the surveys via “think-aloud” cognitive interviews with a subgroup of co-researchers. This resulted in various adaptations to specific items and scales, including changes in phrasing for clarity and removal of questions deemed unrelated to the co-researcher role. For example, co-researchers noted that the use of the word “kids” in one item on the Youth Program Quality Survey was not appropriate, given that co-researchers are teenagers or young adults. Therefore, we rephrased this item to better reflect the age of co-researchers. Table 1 presents an overview of the scales, subscales, specific constructs of interest, adaptations to measures, and measurement time points.

**Table 1:** Youth co-researcher survey measures and data collection time points.

Original scale	Construct and domain (subscale)	Adaptations	Baseline	Intermediate	End line
Social Emotional Health Survey	Self-efficacy and belief in self	Used in full	✓		✓
Sociopolitical Control Scale for Youth	Leadership competence	Used in full	✓		✓
Perceived Control Scale	Perceived control (organization level and community level)	Rephrased to define “community” and “organization” Removed 3 items to align with study scope^a^	✓		✓
Measures of community well-being	Flourishing individuals	Six items removed; 1 rephrased to align with study goals	✓		✓
Youth Program Quality Survey	Adult support, empowered skill-building, and positive values	Used in full; rephrased to specify “adults” One item adapted to be appropriate for use with adolescents and young adults^a^		✓	✓
Youth-Adult Partnership Scale	Supportive adult relationships and youth voice in decision-making	Rephrased to specify staff and organizations		✓	✓
Research and Action Self-Efficacy Scale	Participatory behavior	Removed 2 items specific to youth in schools		✓	✓

^a^Adaptations are based on pilot testing and other youth collaborators’ feedback.

#### Youth Co-Researcher Periodic Focus Group Discussions

Approximately each month, focus group discussions will be held with co-researchers. These group discussions will ask co-researchers to reflect on their overall experience and participation with the collaborative IM and implementation processes. These focus groups serve as a touchpoint with co-researchers to gather insights into their experience of the collaboration process, offering them a space to reflect, share feedback, and even propose potential adjustments to improve our approach. Some examples of focus group discussion questions are as follows: What has it been like to be part of this process? What made you feel included and valued in this process? What, if anything, made collaboration or working together challenging? Detailed notes will be taken during each focus group discussion.

#### Youth Co-Researcher Semistructured Exit Interviews

To document co-researchers’ experiences and solicit feedback on our engagement processes, semistructured exit interviews will be conducted with co-researchers when they offboard from their role. Key constructs of interest include general experiences as a co-researcher, perceptions of personal and professional development over time, contributions to the study, team dynamics and communication, key challenges, and opportunities for improvements to youth engagement processes.

#### Youth Advisor Measure: Advisor Survey

To evaluate the advisor engagement process, we will gather feedback from advisors at the end of each feedback opportunity via a brief survey of multiple-choice and open-ended questions. The survey items are based on the Youth Program Quality Survey [83]. The surveys and selected measures will be customized based on the format of the feedback session (group, individual, asynchronous, live, in person, and virtual) and session activities.

### Planned Analysis

Qualitative data (field notes, ethnographic memos, and semistructured exit interviews) will be analyzed using applied thematic analysis procedures, including cycles of memoing, coding, data reduction, and theme development [90,91]. Qualitative data will be managed and coded using Dedoose qualitative analysis software (SocioCultural Research Consultants). A team of coders will begin by memoing the data to develop a codebook and then participate in consensus meetings to refine the codebook, resolve coding discrepancies, and ensure consistent code application. Once the data are coded, coders will develop “code summaries” to reduce the data for theme development. Themes will be initially developed by reviewing code summaries, code frequency and repetition, code co-occurrence, and salience of key concepts. TobExA team members from the academic partner organization will lead analysis and engage members from the community partner organization in key analytic processes, including finalizing themes and interpreting results to inform practice and research [91]. To ensure transparency of our qualitative analysis processes, we will maintain an audit trail to document all coding decisions, including modifications to the codebook, and theme development and interpretation procedures.

Quantitative (survey) data will be summarized using descriptive and inferential statistics as appropriate or possible. At a minimum, we will use descriptive statistics to summarize outcomes for co-researchers at each data point (baseline, intermediate, and end line) and examine individual and group changes over time. Individual scales will be scored in accordance with scoring guidelines. Sociodemographic data will be used to characterize the overall sample of co-researchers and advisors to allow for stratified analyses of data as appropriate (eg, comparing engagement experiences for youth collaborators aged <18 y and >18 y).

Results from quantitative and qualitative data analyses will be periodically (at least quarterly) reviewed by TobExA team members from the community and academic partner organizations. The partners will work together to identify data-driven quality improvement changes to enhance our youth engagement strategies throughout the TobExA project. As appropriate, co-researchers will be provided with summaries of results (aggregated to protect co-researchers’ and advisors’ privacy and confidentiality) and will also be involved in identifying quality improvement strategies to address any needs or concerns based on the data. Results will also be used to generate recommendations to promote equitable youth engagement and collaboration practices in intervention research, which will be shared via academic and nonacademic dissemination channels (eg, scientific manuscripts, practice reports, and briefs).

### Ethical Considerations

Ethics approval for this research was obtained from San Diego State University Institutional Review Board (HS-2023-0248 and HS-2024-0177). We received a waiver of parental consent and written consent and assent for the study. Therefore, all co-researchers provided verbal assent (those under 18) or consent to participate in the evaluation component of the project. The study team has procedures in place to maintain participant privacy and confidentiality.

Co-researchers will receive US $20 per hour of work, with additional stipends to cover transportation and childcare as needed. Hours worked and travel for study-related tasks will be reported weekly, with checks issued by the community partner organization biweekly. Payment for work is not contingent upon completing evaluation activities (eg surveys, interviews).

## Results

### Youth Co-Researchers

As of December 2024, we have onboarded 7 co-researchers (aged <18 y: n=2, 29%; aged >18 y: n=5, 71%). Initial co-researcher–identified interests include gaining experience in administrative tasks, social media and in-person outreach, research (eg, data collection), disseminating research to diverse audiences, and advocacy. Co-researchers have participated in professional development, capacity building, and study-specific activities to date, as shown in Textbox 1 [64,92,93].

In Table 2, we summarize sociodemographic characteristics of 4 co-researchers who have provided data to date. Co-researchers have worked an average of 5.9 (SD 2.7; range 1.5-10) hours per week and have received compensation for their contributions in the form of checks approximately every 2 weeks (and gift cards for transportation, ie, Uber or Lyft, as needed). Data collection for engagement-related outcomes is ongoing and will be reported in future publications as appropriate.

**Professional development, capacity building, and study-specific activities completed by the co-researchers.**

- Co-researchers completed a 3-hour “CIRTification” human research protections training program. CIRTification is an established curriculum developed for community members engaging in research that can be used as an alternative to the Collaborative Institutional Training Initiative human participants research training courses required by most institutional review boards [92].
- Co-researchers completed a now defunct self-paced virtual advocacy trainings through Take Down Tobacco offered through the Campaign for Tobacco-Free Kids.
- Co-researchers completed a Google Workspace training.
- Co-researchers reviewed adapted study overview documents (eg, a youth-friendly version of the funded grant application) and engaged in group discussion regarding the goals and processes of the Tobacco and Cannabis Harm Reduction through Expressive Arts (TobExA) study and ideas for intervention approaches.
- Co-researchers developed advisor recruitment materials (flyers and interest surveys in English and Spanish).
- Co-researchers provided oral feedback and guidance during the initial stages of the intervention mapping process. For example, 57% (4/7) of the co-researchers attended a virtual group meeting with TobExA team members from the community and academic partner organizations to provide feedback on the in-development “logic model of the problem,” one of the first tasks in intervention mapping [64,93].
- Co-researchers reviewed and provided written and oral reflections in response to brief prompts regarding the appropriateness and overall utility of available youth substance use prevention and intervention programs. Initially, we asked the co-researchers to locate these programs themselves through web searches; however, based on feedback from co-researchers, we amended this task so that the youth engagement liaisons first located and condensed information about existing programs into a 1- to 2-page document that could then be reviewed by the co-researchers.
- Co-researchers selected and piloted “take-home” expressive arts activities to consider for inclusion in the TobExA intervention.

**Table 2:** Youth co-researcher sociodemographic characteristics (n=4).

Sociodemographic characteristics		Values
Age (y), mean (SD)		19.3 (2.3)
**Gender identity, n (%)**		
	Woman	3 (75)
	Gender fluid	1 (25)
**Sexual orientation, n (%)**		
	Heterosexual	1 (25)
	Fluid	1 (25)
	Bisexual	1 (25)
	Queer	1 (25)
**Education, n (%)**		
	Less than high school	2 (50)
	High school graduate or GED^a^	1 (25)
	Some college	1 (25)
**Race or ethnicity, n (%)**		
	Black or African American	1 (25)
	Hispanic or Latino	1 (25)
	Other race or ethnicity	2 (50)
**Transgender identity, n (%)**		
	No	4 (100)
**Homelessness or housing instability (past 6 months; not mutually exclusive), n (%)**		
	Shared the housing of other persons	2 (50)
	Lived or stayed in cars, parks, public spaces, abandoned buildings, substandard housing, or bus or train stations	1 (25)
	Other living situation not listed	1 (25)
	Prefer not to answer	1 (25)

^a^GED: General Educational Development.

### Youth Advisors

As previously noted, we engaged members of the community partner YAB at the beginning of the study to guide the development of our youth engagement and collaboration approach. During the initial engagement with the YAB to develop our youth collaborator engagement processes, we piloted the advisor survey to refine the survey measure and evaluate the strategies we used to interact with members of the YAB (such that we could adjust strategies for future youth engagement). Of the 5 YAB members who completed the survey, 4 (80%) “strongly agreed” and 1 (20%) “strongly disagreed” with the statement “the TobExA team listened to what I had to say*.*” All “agreed” or “strongly agreed” that they “felt comfortable giving [their] opinion out loud,” “enjoyed this activity,” and that “there is a good balance of power between YAB members and [academic and community partners] in these meetings.” These results suggest that using similar collaboration strategies with advisors will allow them to feel heard and comfortable sharing opinions and feedback regarding the TobExA intervention. Piloting these measures allowed us to identify 1 question that should be removed before implementing the survey with the advisors, as it was deemed unclear and repetitive.

Additional TobExA advisors were engaged from May to July 2025 (n=5). Some advisors provided asynchronous written feedback via email over multiple instances (n=2, 40%). Topics addressed through this approach included feedback on recruitment strategies, facilitation techniques, general program content, and appropriate participant remuneration. Other advisors attended a one time in person meeting to provide feedback on potential program activities (n=4, 80%).

## Discussion

### Anticipated Findings

The use and effectiveness of youth engagement strategies in the context of intervention research (eg, co-design) have been underreported and underevaluated in the existing literature. To fill this gap, we have developed youth engagement strategies to promote authentic, equitable, and sustained youth collaboration throughout the TobExA intervention research process. These strategies include flexible options for engagement based on youth interests, availability, and personal and professional goals; fair compensation for contributions; tangible support for transportation and childcare needs; access to technology and a physical work space; leadership and professional development opportunities; and guidance and support from community and academic partner organizations’ staff.

On the basis of our preliminary findings, we have identified several potential barriers to and facilitators of effective engagement of youth collaborators in the TobExA study. As we complete our study aims, we will leverage these insights to continuously improve and sustain effective youth engagement approaches. We believe these learnings may be useful to other researchers who aim to incorporate similar youth collaboration goals into their studies.

### Challenges

#### Financial Compensation

Concerns regarding equitable compensation for TobExA youth collaborators were a significant focus in initial meetings with the community partner YAB, with YAB members raising concerns about the amount, frequency, and form of compensation (ie, concerns regarding compensation in the form of gift cards vs cash or check stipends). We were pleased to develop a plan that addressed the concerns raised by the YAB members; however, we did not sufficiently outline and create a detailed protocol of the process for compensation and specific roles of each partner organization in ensuring that co-researchers received payment for their contributions. This lack of a formal process led to confusion and, in turn, delays in payment early in the study. In addition, although both the community and academic partner organizations have existing payment structures to compensate collaborators and research participants, co-researchers were not initially onboarded as “employees” or “participants” at either organization, given their unique role on TobExA. This further complicated payment processes, causing some delays in co-researchers receiving payments for their contributions. These challenges led to the community and academic partners working together to delineate a specific protocol for tracking and communicating co-researchers’ hourly contributions to the study, thereby decreasing confusion or frustrations among co-researchers and other TobExA team members and improving the timeliness of payment. This experience is aligned with many challenges regarding compensation for youth involved in community-engaged research [94] and emphasizes the importance of establishing clear processes for compensation before engaging youth collaborators.

#### Navigating Professional Boundaries

Another challenge relates to navigating and maintaining professional boundaries between co-researchers, youth engagement liaisons, and other members of the TobExA team who work with the co-researchers outside of the TobExA study, that is, as service providers at the community partner organization. For example, there have been some occasions in which a co-researcher contacted a specific TobExA team member from the academic partner organization with a request. If the co-researcher was unsatisfied with the response, they would then contact a TobExA team member from the community partner organization with the same request in an attempt to get a different response. Alternatively, a co-researcher would contact one TobExA team member from each of the partner organizations (community and academic) with the same request. If or when responses differed or partners deferred to the other, the co-researcher would become understandably frustrated. This led to duplicative staff efforts (2 individuals attempting to respond to the same request) and the need for lengthy discussion among TobExA team members regarding relatively minor requests.

TobExA team members from the community partner organization have likened this to “staff splitting,” a phenomenon that occurs in health and social services when clients with multiple staff or clinicians responsible for their care engage in actions that create division or polarization among staff in order to influence decisions around their care [95]. While a frequent example of staff splitting in the literature relates to clients seeking more leniency in their treatment plans [96], our experiences have been related to co-researcher expectations regarding responsibilities and processes related to remuneration. Although “staff splitting” has been a new experience to TobExA team members from the academic partner organization, those from the community partner organization (ie, service providers and administrators) have explained that this is a relatively common experience in the provision of social services to youth (and may be aligned with the youth’s developmental stage). They have provided guidance to the TobExA youth engagement liaisons on how to mitigate similar issues in the future.

Relatedly, there have been challenges regarding communication (eg, timely responses to messages), meeting attendance, and completing assigned activities on the part of the co-researchers. This is expected given that for many co-researchers, this is the first time working in a professional work setting. Our ability to overcome these challenges has been somewhat complicated by team members from the community and academic partner organizations having different ideas for resolution. TobExA team members from the academic partner organization were initially very cautious about enforcing specific expectations regarding communication, attendance, and transparency, fearing such an approach would alienate co-researchers and lead to disengagement. In contrast, several TobExA team members from the community partner organization and even some of the co-researchers have stressed the need to foster and uphold basic professional standards for the co-researcher role, such as responses to communications and requests within an agreed-upon period. Indeed, TobExA team members from the community partner organization encouraged those from the academic partner organization to view the co-researcher role as an important opportunity for youth with limited resources and access to professional development opportunities to develop professional skills in a supportive environment that could benefit them in future jobs and in navigating their transition into a stable adulthood.

#### Competing Priorities of Youth Collaborators

We have experienced challenges gathering all co-researchers for collaborative working sessions, given their many competing priorities, as all co-researchers are in school or have other work commitments, and many have specific family commitments, such as parenting. This is a challenge we are accustomed to navigating in community-academic research efforts in which team members have many competing priorities and limited availability. Much of the co-researchers’ tasks on TobExA are designed to be conducted remotely; however, there is a need for at least some collaborative working sessions and these competing priorities and busy schedules have made it challenging for all co-researchers and key members of the TobExA youth engagement team to meet. We have addressed this challenge through a flexible meeting schedule and increasing support for co-researchers to attend meetings in person (eg, additional transportation support).

#### Legitimization of the Co-Researcher Role

Other challenges regarding the organizational legitimization of the co-researcher role have occurred to date. For example, co-researchers are required to complete administrative forms to officially “volunteer” (ie, work) with the academic partner organization and to obtain an institution-specific email address. These forms request information that may be challenging for some co-researchers to provide, such as a permanent address or government ID verification. This was further complicated when our study staff learned that the academic partner organization requires volunteers to be aged ≥18 years. To address these barriers, youth engagement liaisons worked with co-researchers to complete necessary forms. For those aged <18 years, the community partner organization issued an email address. While obtaining “official” status with an academic partner organization may be beneficial to youth collaborators, it is important to critically examine how institutional barriers (particularly at academic institutions) may act as a form of gatekeeping, restricting youth’ ability to engage in research [97].

#### Technological Barriers

Many of the co-researchers have experienced technological barriers to participation. For example, a lack of reliable access to computers has made it difficult for some co-researchers to complete self-paced training, as they had to complete the training on their mobile phones or come in-person to a provided workspace to complete it. In addition, at least 1 co-researcher lost access to their mobile phone during their time collaborating on the study, delaying their communication with the youth engagement liaisons and other TobExA team members. Although both the community and academic partners provide access to computers with internet at their respective organizations, co-researchers have experienced challenges coming to these locations during operational hours. To address this issue, we have worked with co-researchers to identify ideal in-person working times and ensured TobExA team members are available to be on-site and provide oversight and guidance for assigned tasks during these times.

Researchers who aim to collaborate with youth, including youth experiencing homelessness and housing instability, should anticipate and prepare to address technological barriers to participation, which may include the provision of computer devices for temporary use, free software programs [98], and nondigital approaches [99]. Researchers should develop clear protocols to address broken or lost devices and be mindful of related organizational requirements.

### Facilitators of Youth Engagement

We have identified several facilitators of youth engagement to date. For example, we have found that engaging multiple direct-service providers from the community partner organization in the youth engagement process has strengthened and facilitated co-researcher recruitment. Specifically, staff with established rapport with individual youth have helped us to recruit highly engaged co-researchers. Relatedly, cocreating the co-researcher and advisor roles with the community partner YAB helped us ensure that each position was appropriate and appealing to potential youth collaborators and resulted in us recruiting multiple YAB members to become co-researchers for the TobExA study.

Another facilitator of youth engagement and collaboration has been tailoring each co-researcher’s research and training tasks to their individual goals and interests. On the basis of interviews with prospective co-researchers and initial onboarding meetings, we have identified customized training opportunities and tasks for each co-researcher. For example, some co-researchers indicated an interest in learning more about the use of social media in research and were subsequently assigned tasks related to reviewing and evaluating existing social media accounts related to substance use prevention to inform the TobExA intervention.

### Strengths and Limitations

The quality of our protocol must be considered in the context of its strengths and limitations. A key strength is that our youth engagement approach is embedded within a larger community-based participatory research study that builds upon a multiyear community-academic partnership. This infrastructure allows for our learnings to be implemented and sustained within the partnership moving forward. Another strength is our approach to evaluation, including adaptation of validated quantitative measures to assess how youth collaborators may benefit from participation in the study and qualitative measures to document the process and identify opportunities for real-time quality improvement. We are particularly pleased to be able to fairly compensate youth collaborators for their contributions, which aligns with recommendations from the community partner’s YAB and with established best practices of youth engagement. Furthermore, our payment structure provides hourly compensation for the youth’s contributions to the study, with more involved, sustained roles earning greater compensation for their contributions compared to those who engage as advisors. Finally, our adaptable and flexible youth engagement process is a strength, as it allows us to respond in real time to necessary changes or challenges related to engaging youth collaborators.

A key limitation relates to engaging youth who do not primarily speak English or have limited English proficiency. Indeed, TobExA team members from the community partner organization have consistently expressed concerns that youth engagement would not be inclusive of youth who prefer Spanish and seek housing-related services at their organization. While we have developed some materials in Spanish and have plans for increasing engagement of youth who prefer Spanish as co-researchers and advisors, we have yet to engage youth who prefer Spanish specifically (we do have bilingual [English and Spanish] co-researchers on the team). A related limitation is the sociodemographic characteristics of our current co-researchers who are aged 19 years, on average, have completed some college, and are predominantly cisgender young women. Those whom we have yet to sufficiently engage, including youth aged <18 years, young men, and transgender youth, may require different approaches to collaboration due to developmental, social, or gender-specific differences.

In addition, we did not include quantitative measures that explicitly assess the co-researchers’ or advisors’ colearning or codevelopment regarding tobacco- and cannabis-specific prevention or advocacy. We instead selected measures that capture these community-based participatory research principles broadly, including the following: (1) the Research and Action Self-Efficacy Scale, which captures participatory behaviors (eg, “I have spoken with other youth about issues that I want to improve in the community.”) [85]; (2) the Youth-Adult Partnership Scale, which assesses collaborative youth-adult relationships (eg, “There is a good balance of power between co-researchers and SDYS/SDSU staff on the TobExA team.”) [84]; and (3) the Youth Program Quality Survey, which evaluates perceived skill-building opportunities (eg, “I was challenged to think and build new skills.”) [83]. In addition, our qualitative data (semistructured exit interviews and process-related field notes) may reveal whether more specific, tobacco- and cannabis-related learning has occurred. While this approach may limit our understanding of outcome-specific knowledge gains, our measurement plan may be more applicable across content areas. We plan to include more targeted measures of tobacco- and cannabis-related colearning and codevelopment when we pilot the intervention.

Finally, several of the co-researchers engaged to date have served as members of the community partner’s YAB, which has a strong youth engagement and leadership structure. Thus, current and former YAB members who join the TobExA team may be accustomed to collaborating with adult partners and contributing to the development of programs or interventions without the need for extensive capacity building. Youth who have not served on a YAB or in a similar professional capacity may require more support. To foster trust and teamwork among these youth, we plan on implementing strategies to encourage relationship building, including structuring meetings with both co-researchers and advisors to include time for socializing and ice breakers, creating mentor-mentee pairs when appropriate between more senior youth collaborators and those who are newly onboarded and conscientiously creating collaborative groups so that youth have ample opportunity to work with different team members.

### Conclusions

This protocol describes a holistic approach to engaging youth as collaborators (as “co-researchers” and “advisors”) throughout the intervention research process to address tobacco-related disparities among youth experiencing homelessness and housing instability. Our approach may serve as a useful model that can be adapted for other intervention research studies addressing health disparities among young people and research focused on youth and young adults more broadly.

## Appendix

Multimedia Appendix 1 Youth co-researcher and advisor participation during key intervention research stages.

## Abbreviations

IM: intervention mapping
TobExA: Tobacco and Cannabis Harm Reduction through Expressive Arts
YAB: youth action board
