# Supplementary figures and images for: Addressing Tobacco-Related Disparities Among Youth Experiencing Homelessness by Engaging Youth Collaborators in Intervention Research: Protocol for a Multimethod, Community-Based Participatory Research Study
Source: JMIR Res Protoc. 2025 Aug 19;14:e69441. doi: 10.2196/69441 (PMC12405798; doi:10.2196/69441)

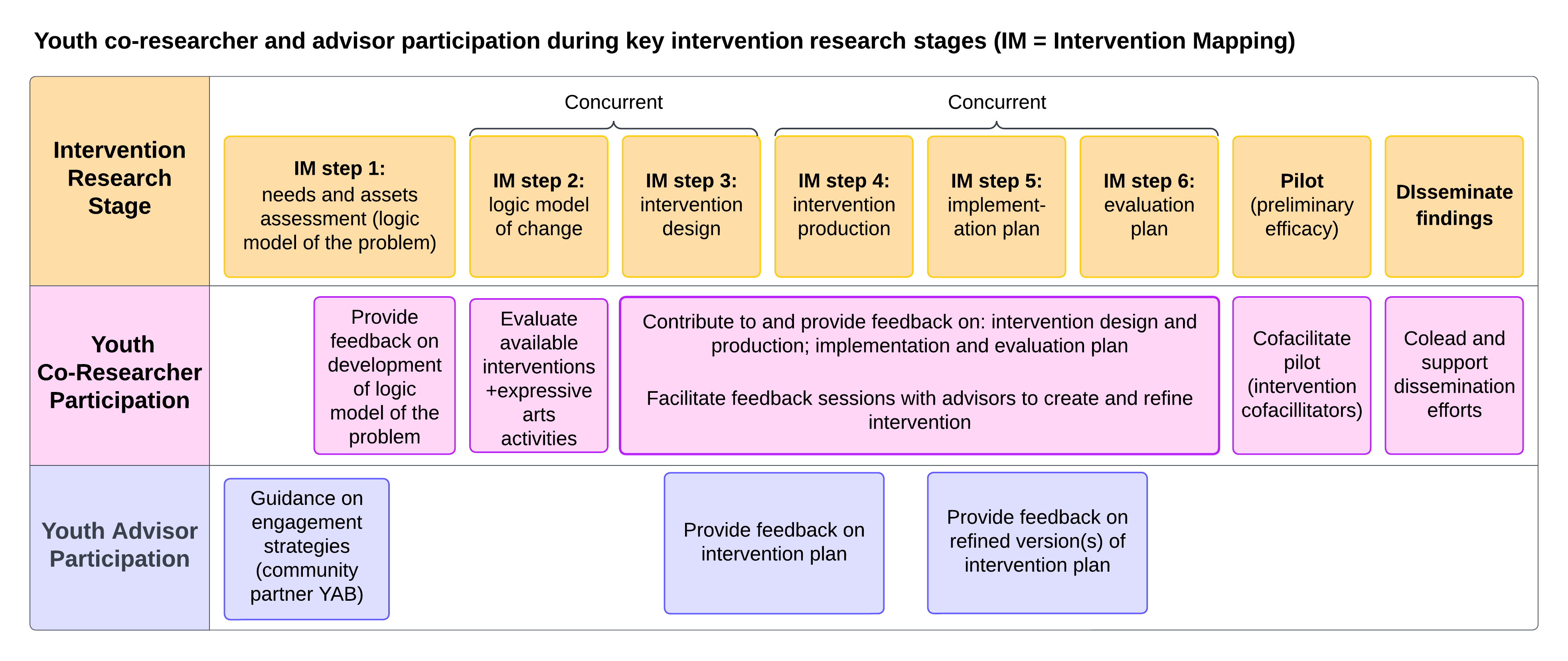

Supplement: Multimedia Appendix 1 [file resprot_v14i1e69441_app1.png]
